# Effects of anesthesia on long-term survival in cancer surgery: A systematic review and meta-analysis

**DOI:** 10.1016/j.heliyon.2024.e24791

**Published:** 2024-01-22

**Authors:** Yaxing Tang, Lele Tang, Yuting Yao, He Huang, Bing Chen

**Affiliations:** Department of Anesthesiology, The Second Affiliated Hospital of Chongqing Medical University, Chongqing, China

**Keywords:** Inhalation anesthesia, Intravenous anesthesia, Cancer surgery, Long-term survival, Meta-analysis

## Abstract

**Backgrounds:**

The association between anesthesia and long-term oncological outcome after cancer surgery remains controversial. This study aimed to investigate the effect of propofol-based anesthesia and inhalation anesthesia on long-term survival in cancer surgery.

**Methods:**

A comprehensive literature search was performed in PubMed, Medline, Embase, and the Cochrane Library until November 15, 2023. The outcomes included overall survival (OS) and recurrence-free survival (RFS). The hazard ratio (HR) and 95 % confidence interval (CI) were calculated with a random-effects model.

**Results:**

We included forty-two retrospective cohort studies and two randomized controlled trials (RCTs) with 686,923 patients. Propofol-based anesthesia was associated with improved OS (HR = 0.82, 95 % CI:0.76–0.88, P < 0.00001) and RFS (HR = 0.80, 95 % CI:0.73–0.88, P < 0.00001) than inhalation anesthesia after cancer surgery. However, these positive results were only observed in single-center studies (OS: HR = 0.76, 95 % CI:0.68–0.84, P < 0.00001; RFS: HR = 0.76, 95 % CI:0.66–0.87, P < 0.0001), but not in multicenter studies (OS: HR = 0.98, 95 % CI:0.94–1.03, P = 0.51; RFS: HR = 0.95, 95 % CI:0.87–1.04, P = 0.26). The subgroup analysis revealed that propofol-based anesthesia provided OS and RFS advantages in hepatobiliary cancer (OS: HR = 0.58, 95 % CI:0.40–0.86, P = 0.005; RFS: HR = 0.62, 95 % CI:0.44–0.86, P = 0.005), gynecological cancer (OS: HR = 0.52, 95 % CI:0.33–0.81, P = 0.004; RFS: HR = 0.51, 95 % CI:0.36–0.72, P = 0.0001), and osteosarcoma (OS: HR = 0.30, 95 % CI:0.11–0.81, P = 0.02; RFS: HR = 0.32, 95 % CI:0.14–0.75, P = 0.008) surgeries.

**Conclusion:**

Propofol-based anesthesia may be associated with improved OS and RFS than inhalation anesthesia in some cancer surgeries. Considering the inherent weaknesses of retrospective designs and the strong publication bias, our findings should be interpreted with caution. Well-designed multicenter RCTs are still urgent to further confirm these findings.

## 1 Introduction

Cancer constitutes an enormous burden worldwide. It was estimated that there were 19.3 million new cancer cases globally and nearly 10.0 million cancer deaths in 2020. The worldwide cancer cases are predicted to increase to 28.4 million in 2040 [1]. Surgical resection is considered the primary treatment for solid malignant tumors. However, cancer recurrence and metastasis after surgery still are the main reasons for cancer-related mortality [2]. Accumulating evidence has shown that anesthetic drugs during surgery may disrupt the delicate balance between tumor invasiveness and host immune surveillance, then contribute to cancer recurrence and metastasis [[3], [4], [5]]. Therefore, it is vital to explore the optimal anesthetics for patients undergoing cancer surgery.

Intravenous and inhalation anesthetics are two commonly used agents for general anesthesia. Studies have reported that inhalation anesthetics might exert suppressive effects on innate and adaptive immunity and enhance the metastatic abilities of cancer cells [[6], [7], [8]]. On the contrary, propofol has been shown to preserve anti-tumor immunity and suppress the growth and survival of cancer cells [[9], [10], [11], [12]]. In addition, laboratory studies have demonstrated that exposure to inhalation anesthetics rather than propofol could induce the upregulation of hypoxia-inducible factor-1α and vascular endothelial growth factor, which promotes cancer cell progression [7,13,14]. These data lead to the hypothesis that propofol might be superior to inhalation anesthetics in cancer surgery. Previous systematic reviews and meta-analyses investigated the association between intravenous and inhalation anesthetics and long-term survival of cancer patients [[15], [16], [17], [18]], however, the conclusions remained inconsistent with insufficient sizes. Subsequently, more studies have been published to address this controversy [[19], [20], [21], [22], [23], [24]]. Therefore, we performed an updated systematic review and meta-analysis to investigate the effect of propofol-based anesthesia and inhalation anesthesia on long-term survival in patients undergoing cancer surgery.

## 2 Methods

This systematic review was conducted according to the methodology described in the Preferred Reporting Items for Systematic Reviews and Meta-Analyses (PRISMA) guidelines (Supplementary Table 1) [25]. The protocol was registered with the International Platform of Registered Systematic Review and Meta-Analysis Protocols (INPLASY202270025, https://inplasy.com/inplasy-2022-7-0025/).

### 2.1 Literature search

As the search strategy shown in Supplementary Table 2, two independent reviewers (YXT and LLT) comprehensively searched eligible trials in the following electronic databases (PubMed, Medline, Embase, and The Cochrane Library) from inception to November 15, 2023, without language restrictions. Furthermore, additional potentially relevant publications were manually searched based on the reference lists of identified trials. Ongoing trials were also searched on the International Clinical Trials Registry Platform (ClinicalTrials.gov).

### 2.2 Inclusion and exclusion criteria

All studies were assessed independently by two reviewers (YXT and LLT). Inclusion criteria: (1) randomized controlled trials (RCTs), non-randomized clinical trials, prospective or retrospective cohort studies that investigated the impact of anesthetic agents on long-term survival during cancer surgery; (2) adult patients undergoing resection of the malignant tumor under general anesthesia; (3) studies that compared propofol-based anesthesia with inhalation anesthesia such as halothane, enflurane, isoflurane, sevoflurane or desflurane; (4) studies that reported overall survival (OS), which was defined as from the date of surgery to the date of death from any cause, or recurrence-free survival (RFS), which was defined as from the date of surgery to the date of first recurrence; (5) studies that reported hazard ratio (HR) and 95 % confidence interval (CI). Exclusion criteria: protocols, comments, reviews, case reports, case-control studies, and studies with insufficient data were excluded. Disagreements were resolved through discussion with a third reviewer (BC).

### 2.3 Study selection

The eligible studies were evaluated independently by two reviewers (YXT and LLT). Initially, the duplicate studies were eliminated by EndNote X9 software (Thomson Reuters, New York). Secondly, the remaining studies were screened through reading titles and abstracts. Finally, full texts were further reviewed for inclusion. Disagreements were reconciled through discussion with a third reviewer (BC).

### 2.4 Data collection

Two reviewers (YXT and YTR) independently extracted relevant data using a standard extraction data form. The following information was extracted from each eligible study: (1) general information included study design, first author’s name, publication year, and country; (2) characteristics of participants included the number of patients, type of surgery, elective or emergency nature of surgery; (3) anesthetic protocol in each group; (4) outcomes included follow-up time, HR and 95 % CIs of reported outcomes. We would contact the original authors by email if relevant data were unavailable. Disagreements were reconciled through discussion with a third reviewer (BC).

### 2.5 Risk of bias in individual studies

Two reviewers (YXT and YTR) independently assessed the quality of the included studies. The randomized control trials (RCTs) were evaluated using the Cochrane risk of bias assessment tool. The evaluation contents included the following aspects: random sequence generation, allocation concealment, blinding of participants and personnel, blinding of outcome assessment, incomplete outcome data, selective reporting, and other biases. Each potential bias was graded as “low risk of bias,” “high risk of bias,” or “unclear risk of bias”. On the other hand, the observational studies were evaluated based on the Newcastle Ottawa Scale (NOS), which includes three domains with a total score of 9 points: selection of subjects (4 points), comparability of the groups (2 points), as well as exposure and outcome ascertainment (3 points). Studies with a NOS score ≥7 were considered to have a lower risk of bias [26]. In case of disagreements, a consensus was reached through discussion with a third reviewer (BC).

### 2.6 Statistical analysis

Review Manager 5.4 software (Cochrane, UK) and Stata 16 (StataCorp LLC, College Station, TX) were used to conduct this meta-analysis. We used the adjusted HRs when both unadjusted and adjusted HRs were provided. To maintain inhalation anesthesia as a reference group, we inverted the HRs and 95 % CIs where intravenous anesthesia was reported as a reference group. Heterogeneity was assessed through *P* and I^2^. Meta-analyses were performed using the random-effects model as we have identified clinical and methodological heterogeneity among studies. To explore the underlying origins of heterogeneity, subgroup analyses were performed based on different types of study centers, cancer surgery, inhalation anesthetics, and statistical models. Publication bias was evaluated using Funnel plots and Egger’s test. P values < 0.05 were considered statistically significant.

## 3 Results

### 3.1 Study selection

**Fig. 1:**
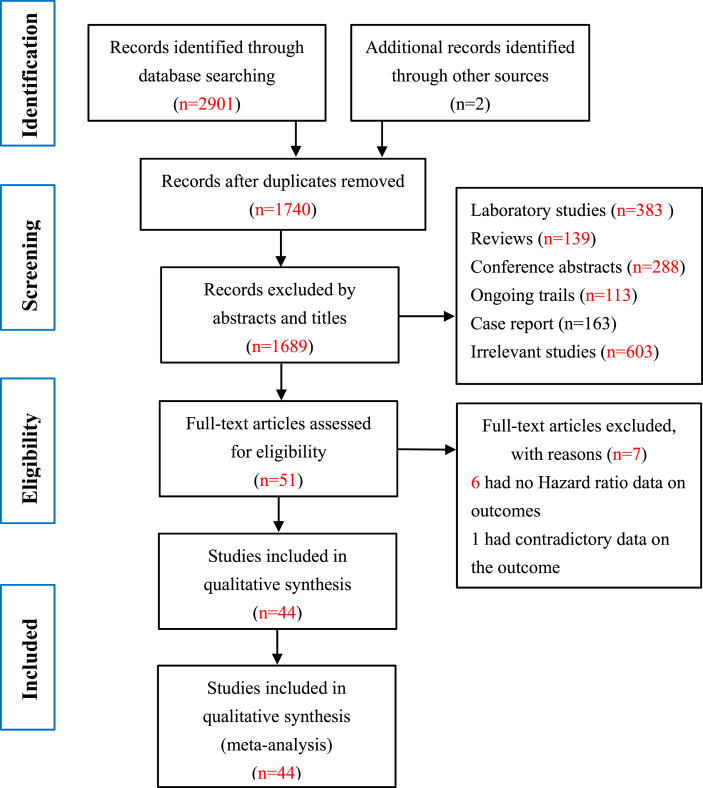
Flow chart of included and excluded studies.

The initial search retrieved 2901 records. Two additional studies were identified from the reference lists. After removing duplicates, 1740 records remained. Of these, 1689 records were excluded through reading titles and abstracts. We further reviewed the full text of the remaining 51 records, the HR data about the outcome was unavailable in 6 studies [[27], [28], [29], [30], [31], [32]], and 1 study had contradictory data on the outcome [33]. Hence, 44 studies (42 retrospective cohort studies and 2 RCTs) met the eligibility criteria (details of the flow chart are shown in Fig. 1). Furthermore, we searched the clinical trial registry platforms and identified nine ongoing RCTs.

### 3.2 Study characteristics

Highlighted characteristics of included studies are presented in Table 1. Forty-two retrospective cohort studies and two RCTs with a total of 686,923 patients were included in this study [[19], [20], [21], [22], [23], [24],[34], [35], [36], [37], [38], [39], [40], [41], [42], [43], [44], [45], [46], [47], [48], [49], [50], [51], [52], [53], [54], [55], [56], [57], [58], [59], [60], [61], [62], [63], [64], [65], [66], [67], [68], [69], [70], [71]]. Thirty-five trials were single-center studies [19,[21], [22], [23],[34], [35], [36], [37], [38], [39], [40], [41], [42], [43], [44], [45], [46], [47], [48],[50], [51], [52], [53],[55], [56], [57], [58],^60^,[62], [63], [64], [65], [66], [67],^69^], nine trials were multicenter studies including four multicenter [49,59,70,71] and five national studies [20,24,54,61,68]. Types of cancer surgery and inhalation anesthetics varied between studies. In total, two studies enrolled participants undergoing surgery for glioblastoma [47,56], one study for oral cancer [63], nine studies for breast cancer [34,37,42,45,49,53,59,61,71], five studies for non-small cell lung cancer [38,55,60,65,69], two studies for esophageal cancer [36,68], four studies for gastric cancer [40,44,50,57], five studies for colorectal cancer [34,39,48,54,62], four studies for hepatobiliary cancer [19,41,43,51], two studies for pancreatic cancer [52,64], two studies for gynecological cancer [21,22], two studies for urological cancer [23,58], one study for osteosarcoma [66], and six studies for various cancers [20,24,35,46,68,70]. For inhalation anesthetics, seventeen studies used sevoflurane to maintain anesthesia [19,21,34,38,40,[47], [48], [49],^53^,^59^,[62], [63], [64], [65],^67^,^70^,^71^], nine studies used desflurane [22,39,[41], [42], [43],^50^,^52^,^56^,^66^] and eighteen studies administered various inhalation anesthetics [20,23,24,[35], [36], [37],[44], [45], [46],^51^,^54^,^55^,^57^,^58^,^60^,^61^,^68^,^69^]. Twenty-six studies included elective surgery [19,21,22,36,[38], [39], [40], [41],^43^,^44^,[46], [47], [48],[50], [51], [52], [53],^56^,^60^,[62], [63], [64], [65], [66], [67],^71^], a part of the participants in three studies undergoing emergency surgery [20,34,54], the remaining fifteen studies did not specify the elective or emergency nature of surgery [23,24,35,37,42,45,49,55,[57], [58], [59],^61^,[68], [69], [70]]. Characteristics of ongoing trials comparing the effects of propofol-based anesthesia and inhalation anesthesia on long-term prognosis of cancer patients are summarized in Supplementary Table 3.

**Table 1:** Characteristics of included studies.

Study	Country	Study design	Study period	Sample size Propofol- based anesthesia Inhalation anesthesia		Cancer type	Property of surgery	Follow up time Propofol-based anesthesia Inhalation anesthesia		Interventions Propofol- based anesthesia Inhalation anesthesia	
Enlund et al. 2014-Colon [34]	Sweden	Retrospective cohort study	1997–2010	179	516	Colon cancer	Emergency and elective	1 or 5 year	1 or 5 year	Propofol	Sevoflurane
Enlund et al. 2014-Rectal [34]	Sweden	Retrospective cohort study	1997–2010	104	202	Rectal cancer	Elective	1 or 5 year	1 or 5 year	Propofol	Sevoflurane
Enlund et al. 2014-Breast [34]	Sweden	Retrospective cohort study	1997–2010	620	1217	Breast cancer	Elective	1 or 5 year	1 or 5 year	Propofol	Sevoflurane
Wigmore et al. 2016 [35]	United Kingdom	Retrospective cohort study	2010–2013	3714/2607^a^	3316/2607^a^	Cancer of breast Gastrointestinal Genitourinary Gynecological Sarcoma origin	NR	2.51 years Median	2.91 years Median	Propofol	Sevoflurane Isoflurane
Jun et al. 2017 [36]	South Korea	Retrospective cohort study	2005–2015	731/439^a^	191/166^a^	Esophageal cancer	Elective	41.1 months Median	25.9 months Median	Propofol	Desflurane Isoflurane Sevoflurane
Kim et al. 2017 [37]	South Korea	Retrospective cohort study	2005–2010	56	2589	Breast cancer	NR	70.1 months Mean	70.1 months Mean	Propofol	Sevoflurane Desflurane Isoflurane Enflurane
Oh et al. 2018 [38]	South Korea	Retrospective cohort study	2003–2012	749/181^a^	194/181^a^	Non-Small Cell Lung Cancer	Elective	5 years Minimum	5 years Minimum	Propofol	Sevoflurane
Wu et al. 2018 [39]	China, Taiwan	Retrospective cohort study	2005–2014	657/579^a^	706/579^a^	Colon cancer	Elective	3.7 years Median	3.2 years Median	Propofol	Desflurane
Zheng et al. 2018 [40]	China	Retrospective cohort study	2007–2012	1506/897^a^	1350/897^a^	Gastric cancer	Elective	43.6 months Median	39.7 months Median	Propofol	Sevoflurane
Lai et al. 2019-A [41]	China, Taiwan	Retrospective cohort study	2005–2014	452/335^a^	492/335^a^	Hepatocellular carcinoma	Elective	NR	NR	Propofol	Desflurane
Huang et al. 2019 [42]	China, Taiwan	Retrospective cohort study	2006–2010	344/296^a^	632/592^a^	Breast cancer	NR	5 years	5 years	Propofol	Desflurane
Lai et al. 2019-B [43]	China, Taiwan	Retrospective cohort study	2005–2014	34/29^a^	36/29^a^	Intrahepatic cholangiocarcinoma surgery	Elective	NR	NR	Propofol	Desflurane
Oh et al. 2019 [44]	South Korea	Retrospective cohort study	2005–2015	816/769^a^	3791/769^a^	Gastric cancer	Elective	1 year	1 year	Propofol	Sevoflurane Desflurane
Yoo et al. 2019 [45]	Korea	Retrospective cohort study	2005–2013	3086/1766^a^	2246/1766^a^	Breast Cancer	NR	67 months Median	53 months Median	Propofol	Enflurane Isoflurane Sevoflurane Desflurane
Hong et al. 2019 [46]	Republic of Korea	Retrospective cohort study	2006–2009	903/729^a^	1304/729^a^	Gastric/stomach, colon, liver, breast, and lung cancers	Elective	5 years	5 years	Propofol	Desflurane Sevoflurane Isoflurane
Dong et al. 2020 [47]	China	Retrospective cohort study	2012–2016	154	140	Supratentorial High-grade Glioma	Elective	12 months Median	12 months Median	Propofol	Sevoflurane
Crone et al. 2020 [48]	Denmark	Retrospective cohort study	2013–2015	483	51	Colorectal cancer	Elective	1199 days Median	1069 days Median	Propofol	Sevoflurane
Enlund et al. 2020 [49]	Sweden	Retrospective cohort study	2006–2012	3096/2298^a^	3209/2298^a^	Breast cancer surgery	NR	NR	NR	Propofol	Sevoflurane
Huang et al. 2020 [50]	China, Taiwan	Retrospective cohort study	2006–2016	190/167^a^	218/167^a^	Gastric cancer surgery	Elective	5 years	5 years	Propofol	Desflurane
Koo et al. 2020 [51]	South Korea	Retrospective cohort study	2003–2013	121	138	Hepatocellular carcinoma	Elective	2 years Maximum	2 years Maximum	Propofol	Inhalation anesthetics
Lai et al. 2020 [52]	China, Taiwan	Retrospective cohort study	2005–2018	72/57^a^	68/57^a^	Pancreatic cancer surgery	Elective	NR	NR	Propofol	Desflurane
Shiono et al. 2020 [53]	Japan	Retrospective cohort study	2008–2012	212/159^a^	814/159^a^	Breast cancer surgery	Elective	59 months Median	59 months Median	Propofol	Sevoflurane
Meng et al. 2020 [19]	China	Retrospective cohort study	2008–2012	208	263	Hepatocellular carcinoma	Elective	5 years	5 years	Propofol	Sevoflurane
Makito et al. 2020 [20]	Japan	Retrospective cohort study	2010–2018	29,337	166,966	Digestive cancer	Emergency and elective	768 days Median	630 days Median	Propofol	Desflurane Sevoflurane Isoflurane
Hasselager et al. 2021 [54]	Denmark	Retrospective cohort study	2004–2018	6322/4347^a^	5238/4347^a^	Colorectal cancer	Elective and acute	54.3 months Median	54.3 months Median	Propofol	Isoflurane Desflurane Sevoflurane
Hayasaka et al. 2021 [55]	Japan	Retrospective cohort study	2010–2016	72	158	Non-small cell lung cancer	NR	54 months Median	64 months Median	Propofol	Sevoflurane Desflurane
Huang et al. 2021 [56]	China, Taiwan	Retrospective cohort study	2008–2018	53/37^a^	50/37^a^	Glioblastoma surgery	Elective	2.5 years Mean	2.1 years Mean	Propofol	Desflurane
Wu et al. 2021 [57]	China	Retrospective cohort study	2009–2016	344/323^a^	2483/645^a^	Gastric cancer	NR	48.8 months Median	48.8 months Median	Propofol	Sevoflurane Desflurane
Pfail et al. 2021 [58]	United States	Retrospective cohort study	2010–2020	126	105	Bladder cancer	NR	4.24 months Median	5.29 months Median	Propofol	Sevoflurane Isoflurane
Takeyama et al. 2021 [21]	Japan	Retrospective cohort study	2006–2018	193/94^a^	94/94^a^	Cervical Endometrial Ovarian cancer	Elective	6.6 years Median	6.6 years Median	Propofol	Sevoflurane
Ttseng et al. 2021 [22]	China, Taiwan	Retrospective cohort study	2009–2014	119/104^a^	165/104^a^	Epithelial ovarian cancer	Elective	5.86 years Median	4.63 years Median	Propofol	Desflurane
Zhang et al. 2021 [59]	China, Taiwan	Retrospective cohort study	2009–2018	1934	1934	Breast intraductal carcinoma	NR	63.5 months Mean	61.8 months Mean	Propofol	Sevoflurane
Watoson et al. 2021 [60]	United Kingdom	Retrospective cohort study	2010–2014	342	404	Non-small cell lung cancer	Elective	3.65 years Mean	3.65 years Mean	Propofol	Isoflurane Sevoflurane
Enlund et al. 2022 [61]	Sweden	Retrospective cohort study	2013–2019	13,873/4658^a^	4801/4658^a^	Breast cancer surgery	NR	33 months Median	33 months Median	Propofol	Desflurane Isoflurane Sevoflurane
Ishiyama et al. 2022 [23]	Japan	Retrospective cohort study	2010–2019	90/70^a^	107/70^a^	Urothelial carcinoma of the upper urinary tract	NR	30.4 months Median	25.6 months Median	Propofol	Sevoflurane Desflurane
Lee et al. 2022 [62]	Korea	Retrospective cohort study	2016–2018	719/717^a^	1852/1410^a^	Colorectal cancer	Elective	42.1 months Median	42.5 months Median	Propofol	Sevoflurane
Miao et al. 2022 [63]	China	Retrospective cohort study	2014–2015	343/302^a^	1004/302^a^	Oral cancer	Elective	62 months Median	69 months Median	Propofol	Sevoflurane
Ren et al. 2022 [64]	China	Retrospective cohort study	2010–2016	114	307	Pancreatic cancer	Elective	17.5 months Median	18.5 months Median	Propofol	Sevoflurane
Yoon et al. 2022 [24]	Korea	Retrospective cohort study	2007–2016	37,063	275,922	Breast, gastric, lung, liver, kidney, colorectal, pancreatic, esophageal, and bladder cancer	NR	1192 days Median	1192 days Median	Propofol	SevofluraneDesflurane Isoflurane Enflurane
Gao et al. 2022 [65]	China	Retrospective cohort study	2013–2016	686/672^a^	1092/672^a^	Non-small-cell lung cancer	Elective	69 months Median	69 months Median	Propofol	Sevoflurane
Sun et al. 2022 [66]	China	Retrospective cohort study	2007–2018	26/22^a^	30/22^a^	Osteosarcoma	Elective	4.66 years Median	2.56 years Median	Propofol	Desflurane
Ma et al. 2023 [67]	China	Retrospective cohort study	2014–2016	147	216	Esophageal cancer	Elective	21.5 months Median	21 months Median	Propofol	Sevoflurane
Oh et al. 2023 [68]	Korea	Retrospective cohort study	2016–2020	58,108/57,685^a^	194,895/57,685^a^	Lung, gastric, colorectal, esophageal, small bowel, liver, pancreatic, and bile duct or gallbladder cancers	NR	90 days or 1 year	90 days or 1 year	Propofol	SevofluraneDesflurane Isoflurane
Seo et al. 2023 [69]	Korea	Retrospective cohort study	2010–2017	980	528	Non-small cell lung cancer	NR	73.7 months Median	73.5 months Median	Propofol	Sevoflurane Desflurane
Cao et al. 2023 [70]	China	Multicentre randomized trial	2015–2017	598	587	Superficial, gastrointestinal, hepatobiliary-pancreatic, lung-esophageal-sternal, genitourinary cancers	NR	43 months Median	43 months Median	Propofol	Sevoflurane
Enlund et al. 2023 [71]	Sweden	Randomized controlled trial	2013–2017	841	829	Breast cancer	Elective	76.7 months Median	76.9 months Median	Propofol	Sevoflurane

a Propensity matched data; NR, no reported; OS, overall survival; RFS, recurrence-free survival.

### 3.3 Risk of bias assessment

Risk of bias assessments of the retrospective studies are displayed in Table 2. Twelve studies received 7 stars, twenty-five studies received 8 stars, and five studies received 9 stars. The quality of all retrospective studies had a low risk of bias. Both the two multicenter RCTs described randomization and allocation concealment, presented complete data, and had no selective reporting. Due to the inherent nature of study intervention, it was not feasible to blind anesthesiologists to group assignment. Nevertheless, the blinding of participants, outcomes assessors, and other team members were reported in two RCTs. As a result, both included RCTs had a low risk of bias in each aspect and were considered high quality.

**Table 2:** Risk of bias assessment based on the Newcastle-Ottawa scale.

Study	Selection (Maximum score 4)	Comparability (Maximum score 2)	Exposure (Maximum score 3)	Total NOS score (Maximum 9)
Enlund 2014 [34]	**4**	**1**	**2**	**7**
Wigmore 2016 [35]	**3**	**2**	**2**	**7**
Jun 2017 [36]	**4**	**2**	**2**	**8**
Kim 2017 [37]	**4**	**1**	**2**	**7**
Oh 2018 [38]	**4**	**2**	**3**	**9**
Wu 2018 [39]	**4**	**1**	**2**	**7**
Zheng 2018 [40]	**4**	**2**	**2**	**8**
Lai 2019-A [41]	**4**	**2**	**2**	**8**
Huang 2019 [42]	**4**	**2**	**2**	**8**
Lai 2019-B [43]	**4**	**2**	**2**	**8**
Oh 2019 [44]	**4**	**2**	**2**	**8**
Yoo 2019 [45]	**3**	**2**	**3**	**8**
Hong 2019 [46]	**4**	**2**	**2**	**8**
Dong 2019 [47]	**4**	**1**	**2**	**7**
Grone 2020 [48]	**4**	**1**	**2**	**7**
Enlund 2020 [49]	**4**	**2**	**2**	**8**
Huang 2020 [50]	**4**	**2**	**2**	**8**
Koo 2020 [51]	**4**	**2**	**2**	**8**
Lai 2020 [52]	**4**	**2**	**2**	**8**
Shiono 2020 [53]	**4**	**2**	**2**	**8**
Meng 2020 [19]	**3**	**1**	**3**	**7**
Makito 2020 [20]	**4**	**1**	**2**	**7**
Hasselager 2021 [54]	**4**	**2**	**2**	**8**
Hayasaka 2021 [55]	**4**	**1**	**2**	**7**
Huang 2021 [56]	**3**	**2**	**2**	**7**
Wu 2021 [57]	**4**	**2**	**3**	**9**
Pfail 2020 [58]	**4**	**2**	**1**	**7**
Takeyama 2021 [21]	**4**	**2**	**2**	**8**
Ttseng 2021 [22]	**4**	**2**	**2**	**8**
Zhang 2021 [59]	**4**	**2**	**2**	**8**
Watoson 2021 [60]	**4**	**1**	**2**	**7**
Enlund 2022 [61]	**4**	**2**	**3**	**9**
Ishiyama 2022 [23]	**4**	**2**	**2**	**8**
Lee 2022 [62]	**4**	**2**	**2**	**8**
Miao 2022 [63]	**4**	**2**	**2**	**8**
Ren 2022 [64]	**4**	**2**	**2**	**8**
Yoon 2022 [24]	**4**	**2**	**2**	**8**
Gao 2022 [65]	**4**	**2**	**2**	**8**
Sun 2022 [66]	**4**	**2**	**2**	**8**
Ma 2023 [67]	**4**	**2**	**3**	**9**
Oh 2023 [68]	**4**	**2**	**2**	**8**
Seo 2023 [69]	**4**	**2**	**3**	**9**

### 3.4 Publication bias

**Fig. 2:**
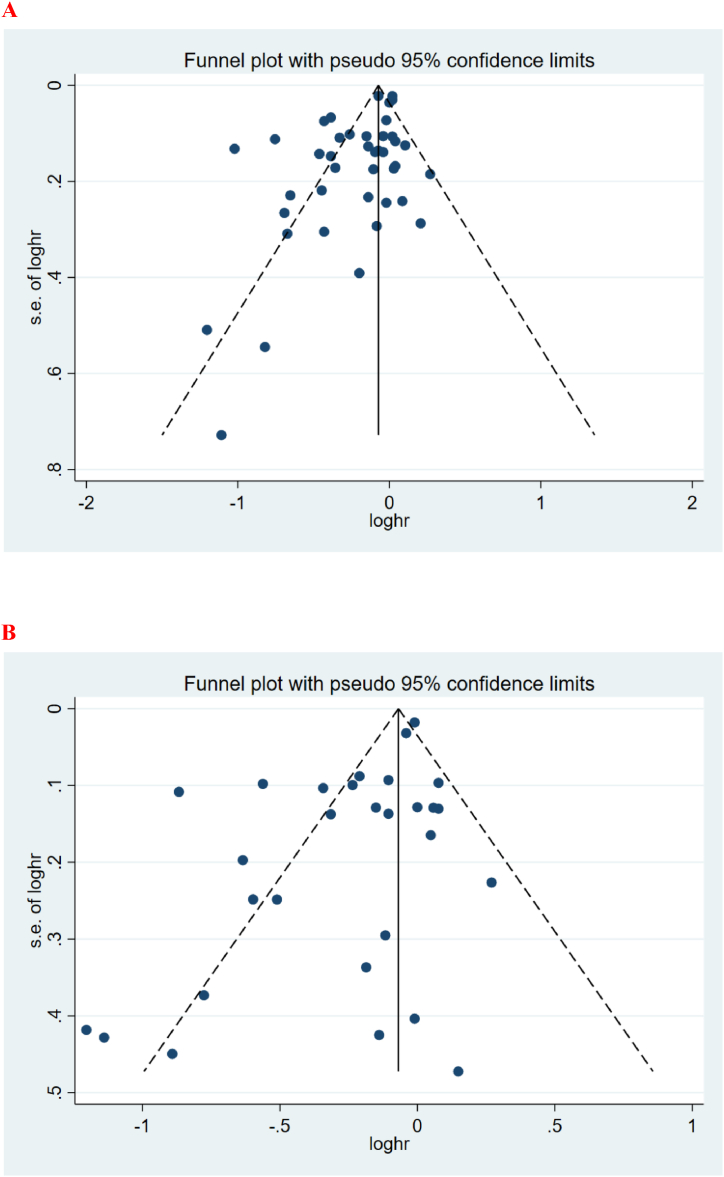
Funnel plots of (A) overall survival and (B) recurrence-free survival.

The asymmetric funnel plot showed that there was a high risk of publication bias both in OS (Fig. 2A) and RFS (Fig. 2B). Moreover, the Egger’s test also indicated a significant publication bias for OS (P = 0.001) and RFS (P = 0.004).

### 3.5 Meta-analysis

#### 3.5.1 Overall survival

**Fig. 3:**
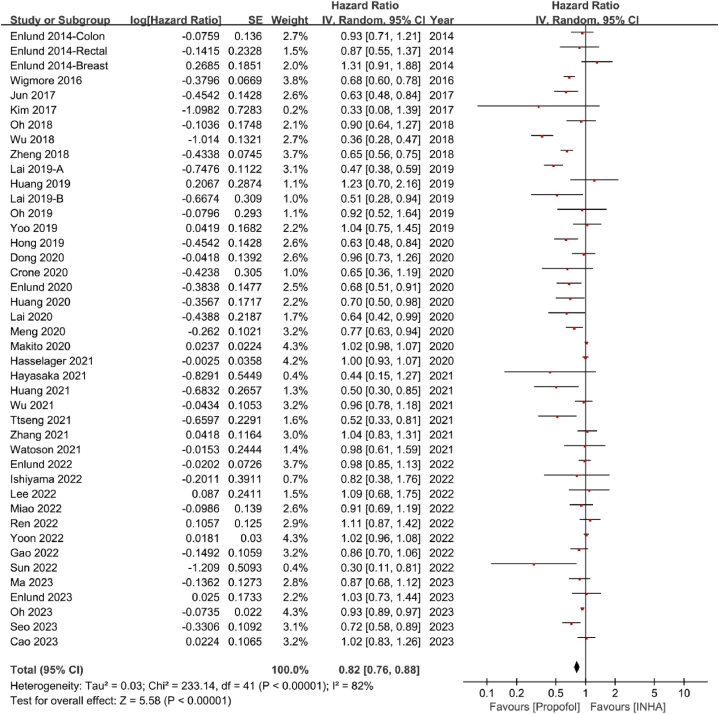
Forest plot of overall survival. HR hazard ratio, CI confidence.

Forty studies reported OS [19,20,[22], [23], [24],[34], [35], [36], [37], [38], [39], [40], [41], [42], [43], [44], [45], [46], [47], [48], [49], [50],^52^,[54], [55], [56], [57],[59], [60], [61], [62], [63], [64], [65], [66], [67], [68], [69], [70], [71]]. When compared with inhalation anesthesia, propofol-based anesthesia was associated with better OS (HR = 0.82, 95 % CI:0.76–0.88, P < 0.00001, I^2^ = 82 %) (Fig. 3). As substantial heterogeneity was observed, we conducted subgroup analyses based on the types of study centers, cancer surgery, inhalation anesthetics, and statistical models.

Nine multicenter studies [20,24,49,54,59,61,68,70,71] and thirty-one single-center studies [19,22,23,[34], [35], [36], [37], [38], [39], [40], [41], [42], [43], [44], [45], [46], [47], [48],^50^,^52^,[55], [56], [57],^60^,[62], [63], [64], [65], [66], [67],^69^] reported on OS. Multicenter studies indicated that propofol-based anesthesia had no advantage in OS compared with inhalation anesthesia (HR = 0.98, 95 % CI:0.94–1.03, P = 0.51, I^2^ = 56 %). In contrast, single-center studies indicated that propofol-based anesthesia had better OS than inhalation anesthesia (HR = 0.76, 95 % CI:0.68–0.84, P < 0.00001, I^2^ = 72 %) (Fig. S1).

Subgroup analysis of different cancer types revealed that propofol-based anesthesia had longer OS than inhalation anesthesia in patients undergoing non-small lung cancer (HR = 0.81, 95 % CI:0.71–0.92, P = 0.002, I^2^ = 0 %), gastric cancer (HR = 0.78, 95 % CI:0.61–0.99, P = 0.04, I^2^ = 70 %) and hepatobiliary cancer surgeries (HR = 0.58, 95 % CI:0.40–0.86, P = 0.006, I^2^ = 81 %). Gynecological cancer (HR = 0.52, 95 % CI:0.33–0.81, P = 0.004) and osteosarcoma (HR = 0.30, 95 % CI:0.11–0.81, P = 0.02) surgeries were also in favor of propofol-based anesthesia, but only one study was included. On the contrary, OS was similar between propofol-based anesthesia and inhalation anesthesia in patients having surgeries of glioblastoma cancer (HR = 0.72, 95 % CI:0.39–1.35, P = 0.31, I^2^ = 78 %), breast cancer (HR = 0.98, 95 % CI:0.85–1.14, P = 0.84, I^2^ = 41 %), oral cancer (HR = 0.91, 95 % CI:0.69–1.19, P = 0.48), colorectal cancer (HR = 0.77, 95 % CI:0.53–1.12, P = 0.17, I^2^ = 91 %), pancreatic cancer (HR = 0.87, 95 % CI:0.51–1.48, P = 0.61, I^2^ = 79 %), urologic cancer (HR = 0.82, 95 % CI:0.38–1.76, P = 0.61), and mixed cancers (HR = 0.94, 95 % CI:0.86–1.02, P = 0.16, I^2^ = 87 %) (Fig. S2).

In terms of different inhalation anesthetics, the pooled data showed that propofol-based anesthesia had better OS compared with sevoflurane (HR = 0.90, 95%CI:0.81–0.99, P = 0.03, I^2^ = 56 %), desflurane (HR = 0.54, 95 % CI:0.43–0.68, P < 0.00001, I^2^ = 64 %) and mixed inhalation anesthetics (HR = 0.89, 95 % CI:0.83–0.96, P = 0.002, I^2^ = 78 %) (Fig. S3).

Twenty-six studies were analyzed by the propensity score matching (PSM) model [22,23,35,36,[38], [39], [40], [41], [42], [43], [44], [45], [46],^49^,^50^,^52^,^54^,^56^,^57^,^59^,[61], [62], [63],^65^,^66^,^68^], while the other twelve studies were analyzed by the non-PSM model [19,20,24,34,37,47,48,55,60,64,67,69]. Propofol-based anesthesia was associated with improved OS than inhalation anesthesia in the PSM model (HR = 0.75, 95 % CI:0.68–0.84, P < 0.00001, I^2^ = 85 %), but not in the non-PSM model (HR = 0.94, 95 % CI:0.87–1.02, P = 0.12, I^2^ = 54 %) (Fig. S4).

#### 3.5.2 Recurrence-free survival

**Fig. 4:**
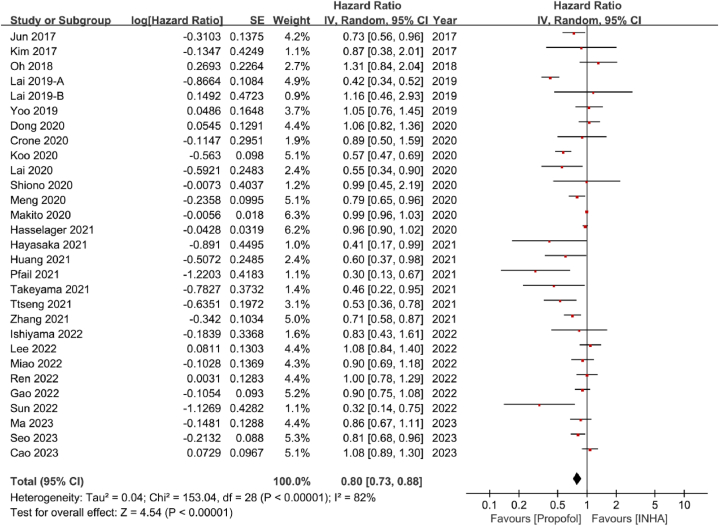
Forest plot of recurrence-free survival. HR hazard ratio, CI confidence.

Twenty-nine studies reported RFS [[19], [20], [21], [22], [23],[36], [37], [38],^41^,^43^,^45^,^47^,^48^,[51], [52], [53], [54], [55], [56],^58^,^59^,[62], [63], [64], [65], [66], [67],^69^,^70^]. Propofol-based anesthesia was associated with better RFS compared with inhalation anesthesia (HR = 0.80, 95 % CI:0.73–0.88, P < 0.00001, I^2^ = 82 %) (Fig. 4). However, there was a significant heterogeneity, consequently, we also conducted subgroup analyses based on different types of study centers, cancer surgery, inhalation anesthetics, and statistical models.

Four multicenter studies [20,54,59,70] and twenty-five single-center studies [19,[21], [22], [23],[36], [37], [38],^41^,^43^,^45^,^47^,^48^,[51], [52], [53], [54],^56^,^58^,[62], [63], [64], [65], [66], [67],^69^] reported on RFS. Regardless of the types of study centers, multicenter studies indicated that propofol-based anesthesia had no advantage in RFS compared with inhalation anesthesia (HR = 0.95, 95 % CI:0.87–1.04, P = 0.26, I^2^ = 75 %). In contrast, single-center studies indicated that propofol-based anesthesia had better RFS than inhalation anesthesia (HR = 0.76, 95 % CI:0.66–0.87, P < 0.0001, I^2^ = 74 %) (Fig. S5).

Subgroup analysis of different cancer types demonstrated that propofol-based anesthesia had better RFS than inhalation anesthesia in patients undergoing esophageal cancer (HR = 0.80, 95 % CI:0.66–0.96, P = 0.02, I^2^ = 0 %), hepatobiliary cancer (HR = 0.62, 95 % CI:0.44–0.86, P = 0.005, I^2^ = 85 %) and gynecological cancer surgeries (HR = 0.51, 95 % CI:0.36–0.72, P = 0.0001, I^2^ = 0 %). For osteosarcoma surgery, only one study was included, which indicated that TIVA was associated with better RFS compared with inhalation anesthesia (HR = 0.32, 95 % CI:0.14–0.75, P = 0.008). On the contrary, RFS was similar between propofol-based anesthesia and inhalation anesthesia in patients having surgeries of glioblastoma cancer (HR = 0.83, 95 % CI:0.48–1.43, P = 0.50, I^2^ = 75 %), breast cancer (HR = 0.84, 95 % CI:0.66–1.08, P = 0.17, I^2^ = 31 %), oral cancer (HR = 0.90, 95 % CI:0.69–1.18, P = 0.45), non-small lung cell cancer (HR = 0.88, 95 % CI:0.70–1.10, P = 0.25, I^2^ = 56 %), colorectal cancer (HR = 0.96, 95 % CI:0.91–1.02, P = 0.23, I^2^ = 0 %), pancreatic cancer (HR = 0.77, 95 % CI:0.43–1.38, P = 0.38, I^2^ = 78 %), urologic cancer (HR = 0.51, 95 % CI:0.19–1.41, P = 0.19, I^2^ = 73 %), and mixed cancers (HR = 1.01, 95 % CI:0.97–1.04, P = 0.70, I^2^ = 0 %) (Fig. S6).

In terms of different inhalation anesthetics, the pooled data showed that propofol-based anesthesia had better RFS compared with desflurane (HR = 0.50, 95 % CI:0.40–0.63, P < 0.00001, I^2^ = 31 %) and mixed inhalation anesthetics (HR = 0.81, 95 % CI:0.71–0.93, P = 0.002, I^2^ = 83 %). On the contrary, there was no significant difference between propofol-based anesthesia and sevoflurane in RFS (HR = 0.92, 95 % CI:0.83–1.02, P = 0.10, I^2^ = 42 %) (Fig. S7).

Seventeen studies were analyzed by PSM model [[21], [22], [23],^36^,^38^,^41^,^43^,^45^,[52], [53], [54],^56^,^59^,^62^,^63^,^65^,^66^], while the other eleven studies were analyzed by non-PSM model [19,20,37,47,48,51,55,58,64]. Both subgroup analyses of PSM and non-PSM models indicated that indicated that propofol-based anesthesia had a better RFS than inhalation anesthesia (HR = 0.76, 95 % CI:0.65–0.90, P = 0.001, I^2^ = 82 %; HR = 0.81, 95 % CI:0.69–0.95, P = 0.01, I^2^ = 81 %) (Fig. S8).

## 4 Discussion

This study investigated whether propofol-based anesthesia or inhalation anesthesia might impact long-term oncological outcomes. We found that propofol-based anesthesia may be associated with improved OS and RFS compared with inhalation anesthesia in all cancer sites, which is partially consistent with the result of an important previous meta-analysis [16]. Chang et al. reported that propofol-based anesthesia was associated with better OS but not RFS than volatile anesthesia in all cancer types [16]. Moreover, growing in vivo and in vitro studies have provided possible explanations to support this claim. First, anesthetics may have different modulatory effects on immune function. Preclinical studies have demonstrated that propofol could protect anti-tumor immunity by preserving the activity and cytotoxicity of circulating NK cells [9,72]. In addition, propofol has been shown to enhance the cytotoxic activity of T-lymphocytes [10], facilitate the activation and differentiation of T helper (Th) cells [73], and retain the Th1/Th2 ratio [74]. By contrast, multiple studies have suggested that inhalation anesthetics may suppress the immune system by reducing NK-cell cytotoxicity [72], declining the Th1/Th2 ratio [6,74], and inducing apoptosis in T lymphocytes [75]. Second, anesthetics may exert direct effects on cancer cells. Propofol has been demonstrated to inhibit the growth and invasion of various cancer cells. This is achieved through different pathways such as increasing the expression of miR-219-5p in hepatocellular carcinoma cells [11], upregulating miR-124-3p.1 and downregulating AKT3 in colorectal cancer [12], suppressing the transcription factor slug in ovarian cancer cells [76], or modulating ERK-VEGF/MMP-9 signaling in Eca-109 esophageal squamous cells [77].

In the subgroup analysis based on the types of study centers, it is worth noting that propofol-based anesthesia had better OS and RFS than inhalation anesthesia only in single-center studies but not in multicenter studies, which was not analyzed in the previous meta-analysis [16]. To achieve a significant difference effect on OS and/or RFS in single-center studies with small sample sizes, an implausibly large effect size seems to be very important [78]. Therefore, such limited external validity may potentially diminish the magnitude of the treatment effects observed in multicenter studies compared to single-center studies. A meta-epidemiological study and a systematic review have reported that single-center studies showed significantly larger treatment effects than multicenter studies [79,80]. Interestingly, when several multicenter studies were combined, propofol-based anesthesia showed no advantage in OS and RFS, which is in keeping with the findings of two multicenter RCTs [70,71]. One RCT [70] found that OS and RFS were comparable between propofol-based and sevoflurane-based anesthesia in mixed cancers. Another RCT [71] detected no significant difference in OS between propofol-based and sevoflurane-based anesthesia for breast cancer surgery. Our conflicting result based on the types of study centers may be attributable to the fact that multicenter studies have larger sample sizes and higher levels of collaboration and cross-validation, while single-center studies may have limited scope and potential biases. Therefore, more prospective multicenter RCTs are warranted to determine the optimal anesthetic choice for cancer surgery. On the other hand, in the subgroup analysis based on the types of cancer, we also found no benefit of propofol-based anesthesia for OS and RFS in mixed cancer and breast cancer surgeries, which is also consistent with the findings of two RCTs. Different cancers may have distinct basic characteristics such as proliferation, invasion, and metastatic abilities, leading to varying survival rates and recurrence. Considering the wide range of cancers and surgical procedures, more well-sized RCTs regarding different types of cancer are needed to further investigate the effects of general anesthetics on long-term survival in cancer surgery.

In addition, we also found differential survival effects based on inhalation anesthetics. Receiving propofol resulted in significantly improved OS and RFS compared to desflurane and mixed anesthetics. However, propofol-based anesthesia only had better OS but not RFS compared to sevoflurane-based anesthesia. Similarly, the impact of inhalation anesthetics on cancer cell biology remains conflicting. Isoflurane could promote the survival and migration of human glioblastoma stem cells and bladder cancer cells [81,82]. Sevoflurane increased invasion and migration in glioblastoma, cervical, and prostate cancer cells [[83], [84], [85]], but not in colorectal cancer, ovarian cancer, colon cancer, breast cancer, and lung cancer cells [[86], [87], [88], [89], [90]]. Analogously, desflurane also showed conflicting results. Desflurane was shown to promote the migration of colorectal cancer and ovarian cancer cells [91,92], but not in glioma cells [93]. These results indicate that the effects of different inhalation anesthetics on cancer cell biology are different.

Our meta-analysis had several limitations. First, the majority of included studies were retrospective observational studies. The retrospective design has an inherent weakness, which cannot be compensated by a meta-analysis of ever so many retrospective studies. Although most retrospective studies performed propensity score matching, there may have been other unpredicted confounding factors and selection bias, making it impossible to prove a causal association. Second, one major weakness of this study is the strong potential for publication bias, which could be attributed to the inclusion of many more single-center studies than multicenter studies. Furthermore, the same research group conducted eight single-center retrospective studies at different times, further increasing the risk of biases. Therefore, our results should be interpreted with caution while considering this limitation. Third, RFS is a somewhat tricky endpoint as most of the included studies did not clearly explain how to define recurrence. Moreover, to be valid, autopsies must be conducted at a frequency of 100 % or close to it, with a rigorous focus on recurrence. Due to the low frequency of autopsies, there may be some inherent uncertainty in the results of RFS. Fourth, cancer staging data is crucial for long-term oncological prognosis, but several included studies lacked this information [24,35,56,68]. The unavailability of tumor staging characteristics can be a significant confounding factor. Finally, surgical techniques and clinical care for cancer patients have improved, resulting in better prognosis. However, some studies included patients over ten years, which may be an additional confounding factor [21,34,52,54,66].

## 5 Conclusions

In conclusion, our current systematic review and meta-analysis indicated that propofol-based anesthesia may have a favorable effect on overall survival and recurrence-free survival compared with inhalation anesthesia in some cancer surgeries. However, considering the inherent weaknesses of retrospective designs and the strong publication bias, caution should be taken when interpreting the results. Well-designed multicenter randomized controlled trials regarding different types of cancer are still needed to clarify the association between general anesthetics and long-term oncological outcomes. Extraordinarily, future studies should be stratified according to different cancer sites, tumor stages, surgical types, and basic characteristics of the patient.

## Funding

This work was supported by Grant [2021]24 from the Kuanren Talents Program of the Second Affiliated Hospital of 10.13039/501100004374Chongqing Medical University.

## Data availability statement

Data included in article/supplementary material/referenced in article.

## CRediT authorship contribution statement

**Yaxing Tang:** Conceptualization, Data curation, Formal analysis, Methodology, Project administration, Writing – original draft. **Lele Tang:** Data curation. **Yuting Yao:** Data curation. **He Huang:** Data curation, Writing – review & editing. **Bing Chen:** Conceptualization, Supervision, Writing – original draft.

## Declaration of competing interest

The authors declare that they have no known competing financial interests or personal relationships that could have appeared to influence the work reported in this paper.
