# Supplementary material for: Effects of anesthesia on long-term survival in cancer surgery: A systematic review and meta-analysis
Source: Heliyon. 2024 Jan 22;10(3):e24791. doi: 10.1016/j.heliyon.2024.e24791 (PMC10839594; doi:10.1016/j.heliyon.2024.e24791)
Supplement: Multimedia component 1 [file mmc1.docx]

**Supplementary Materials**

**Effects of anesthesia on long-term survival in cancer surgery: a systematic review and meta-analysis**

**Supplementary Figure S1:** Subgroup analysis of overall survival according to different research centers

**Supplementary Figure S2:** Subgroup analysis of overall survival according to different cancer types

**Supplementary Figure S3:** Subgroup analysis of overall survival according to different inhalation anesthetics

**Supplementary Figure S4:** Subgroup analysis of overall survival according to the statistical model

**Supplementary Figure S5:** Subgroup analysis of recurrence-free survival according to different research centers

**Supplementary Figure S6:** Subgroup analysis of recurrence-free survival according to different cancer types

**Supplementary Figure S7:** Subgroup analysis of recurrence-free survival according to different inhalation anesthetics

**Supplementary Figure S8:** Subgroup analysis of recurrence-free survival according to the statistical model

**Supplementary Table 1:** PRISMA 2020 checklist

**Supplementary Table 2:** Detailed search strategy

**Supplementary Table 3:** Characteristics of ongoing trials

**Supplementary Figure S1**


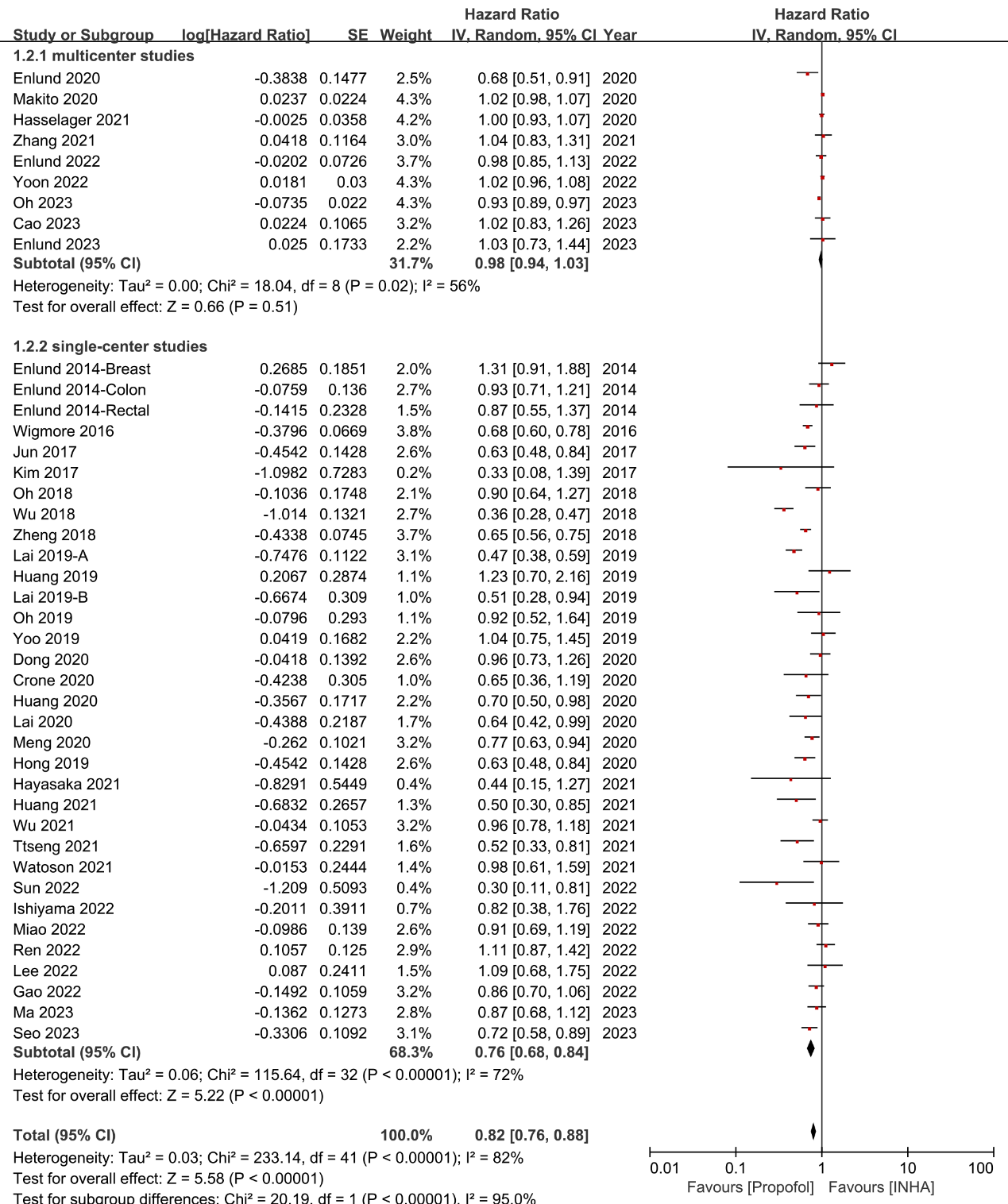


**Supplementary Figure S2**


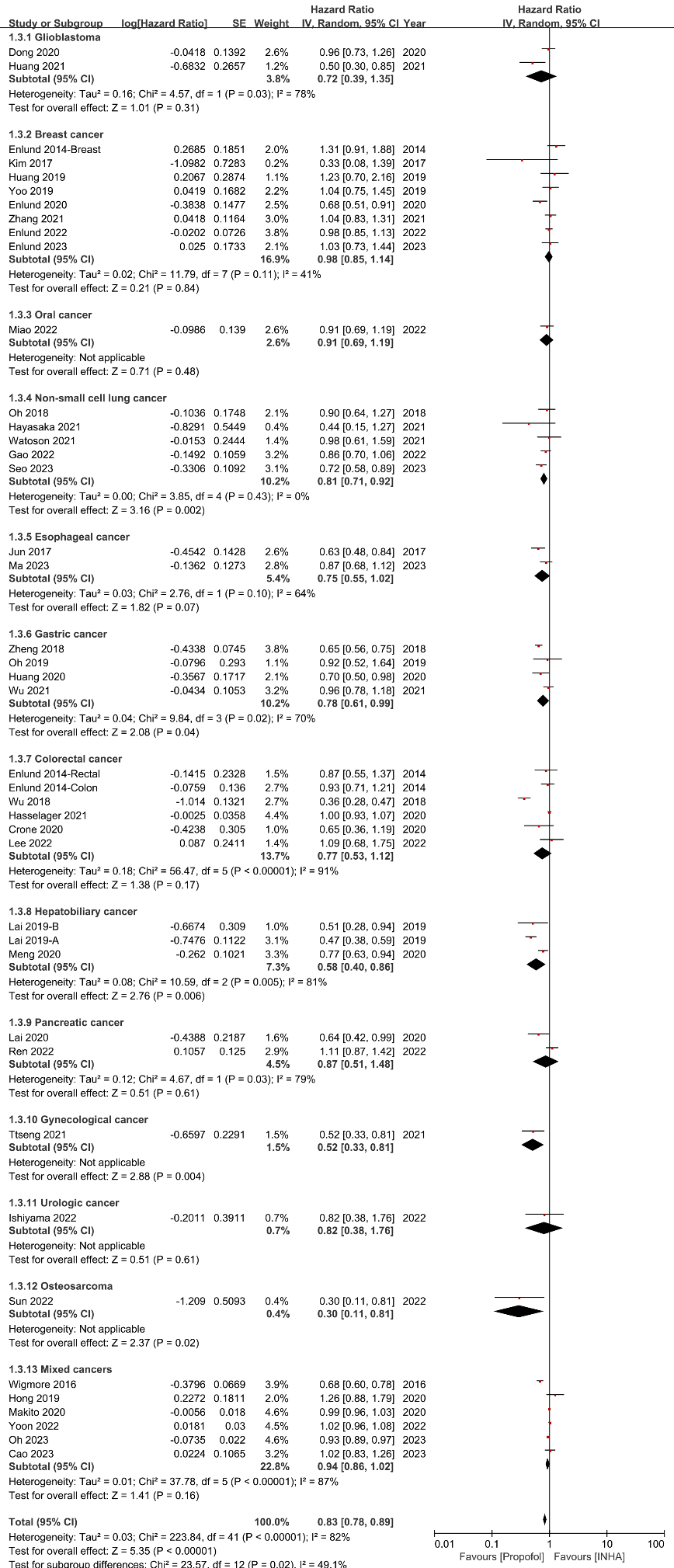


**Supplementary Figure S3**


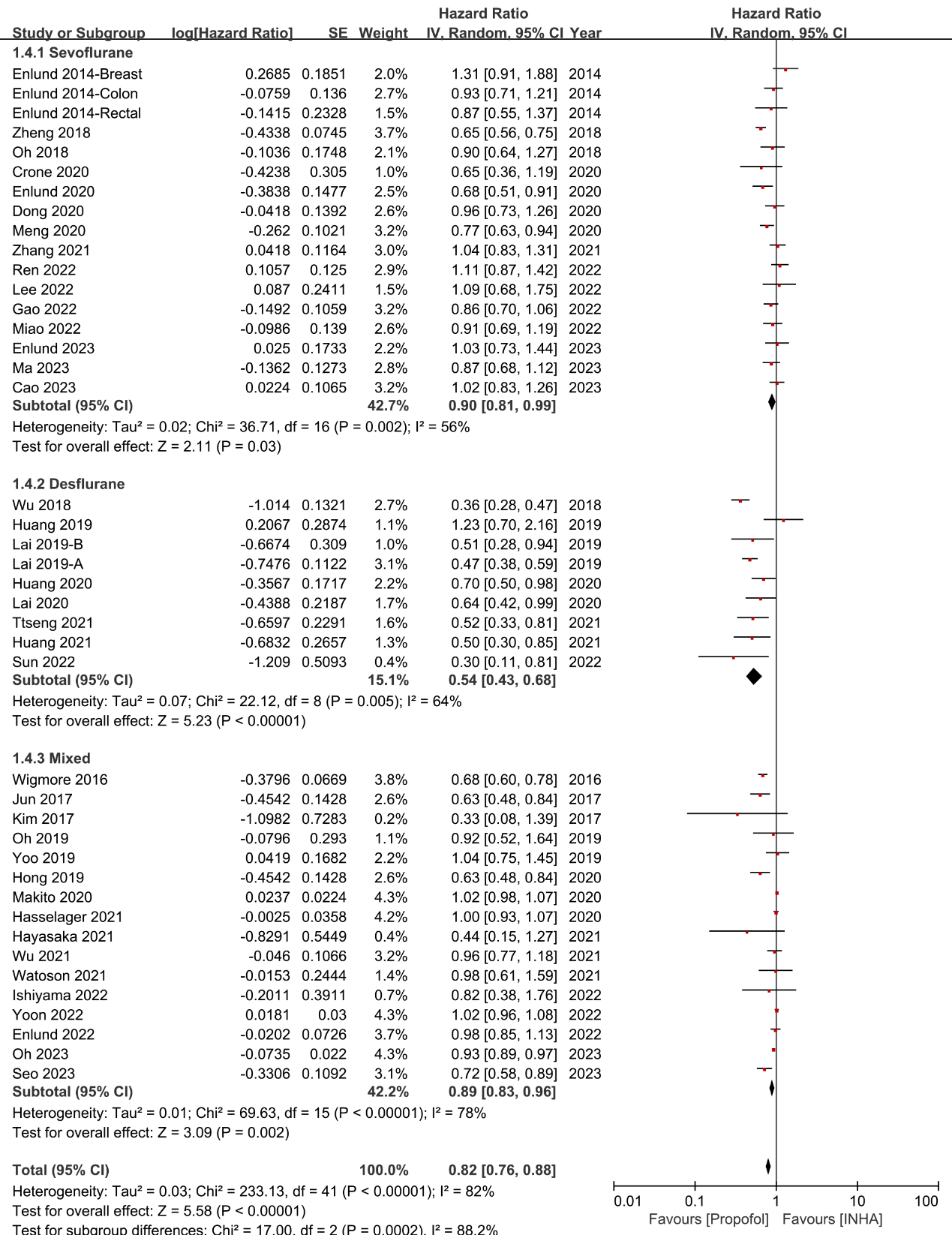


**Supplementary Figure S4**


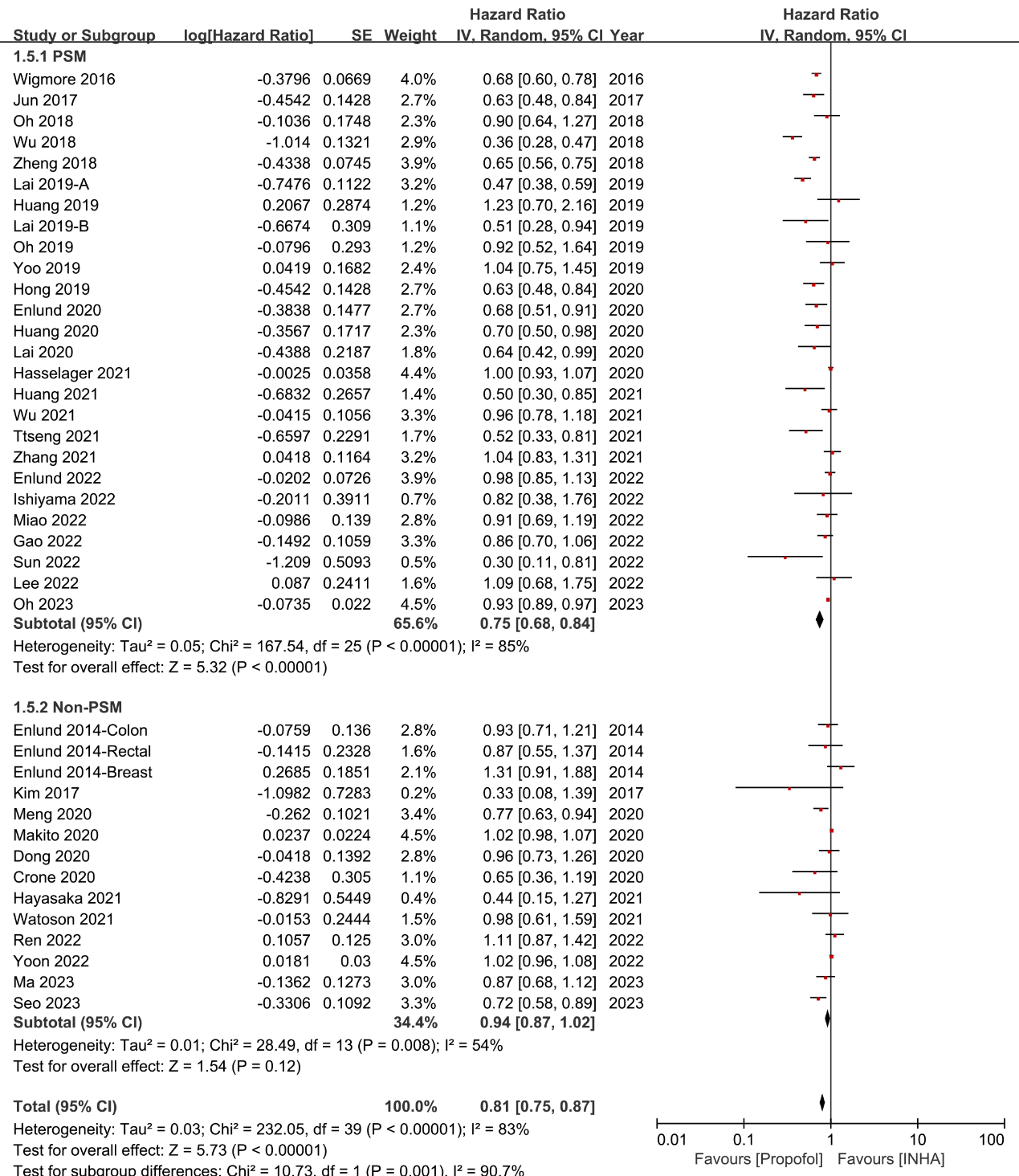


**Supplementary Figure S5**


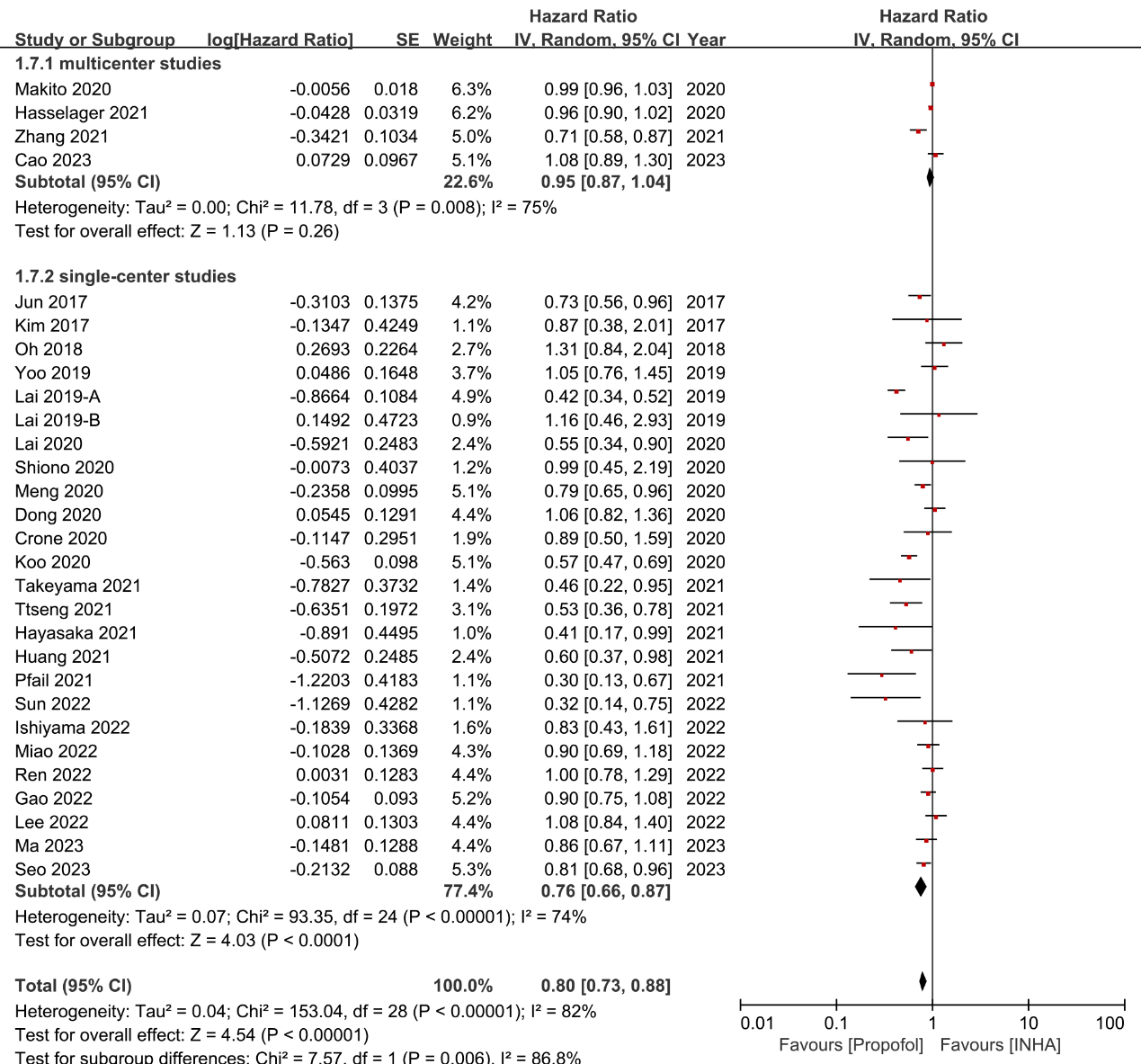


**Supplementary Figure S6**


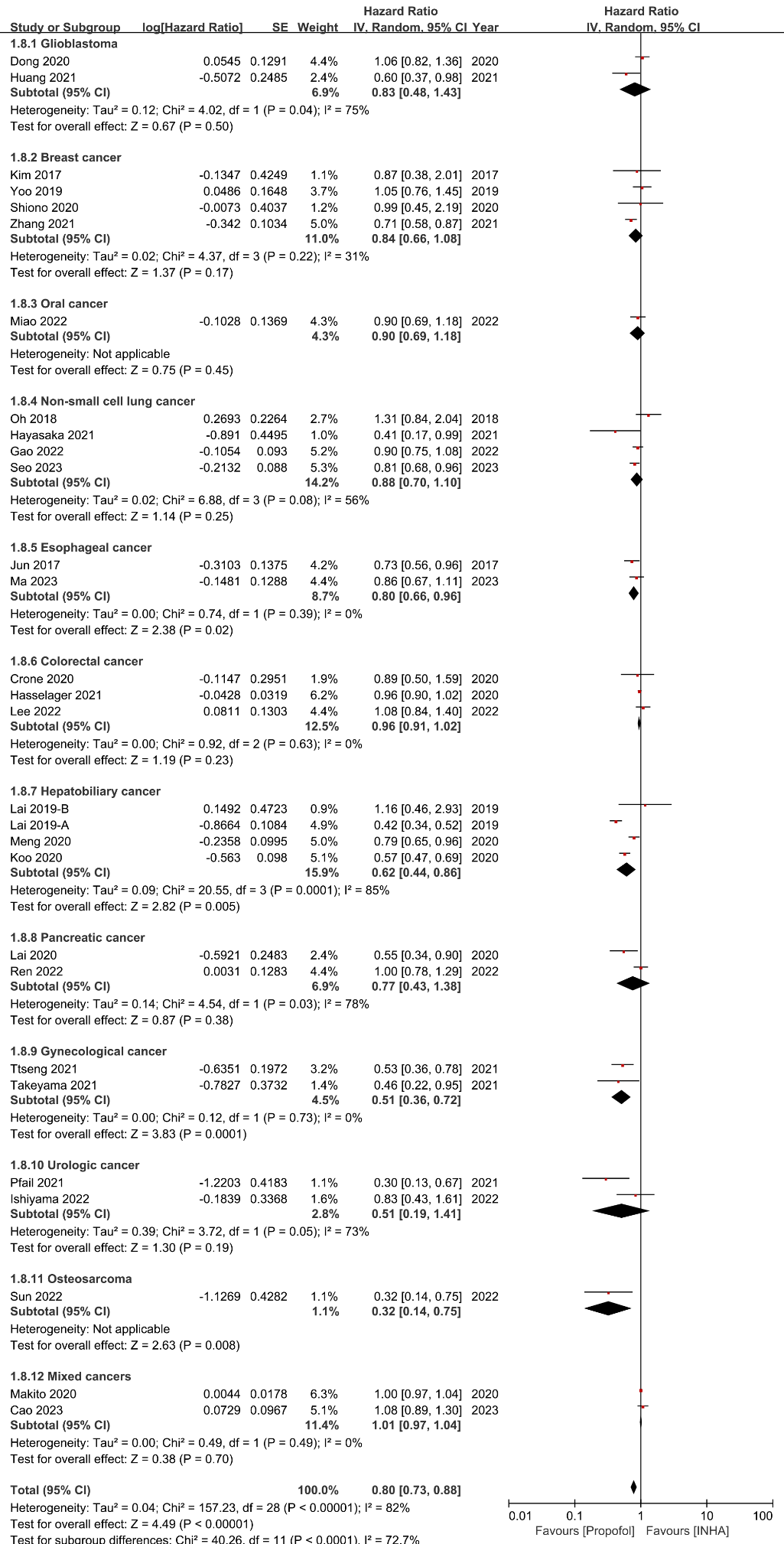


**Supplementary Figure S7**


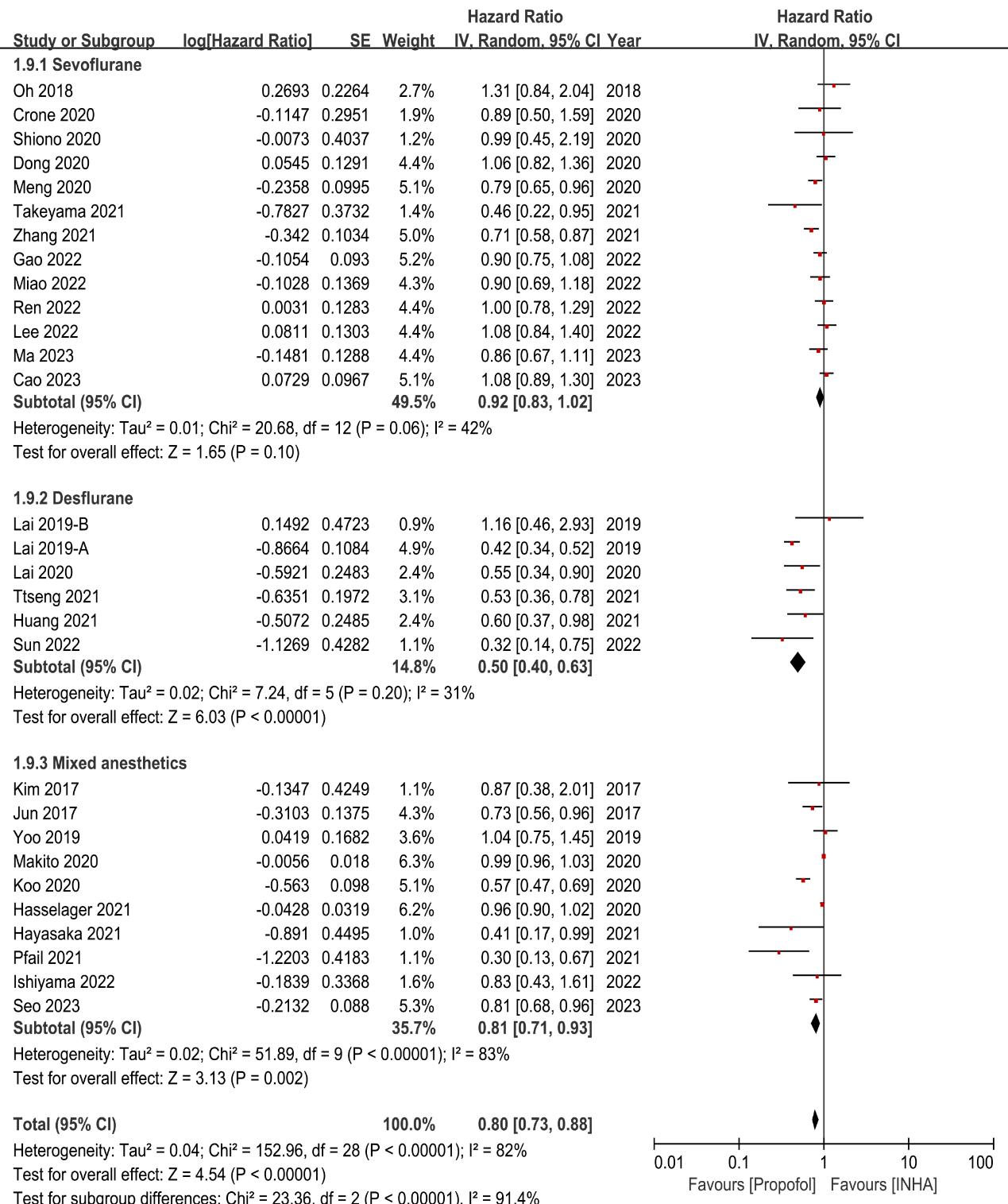


**Supplementary Figure S8**


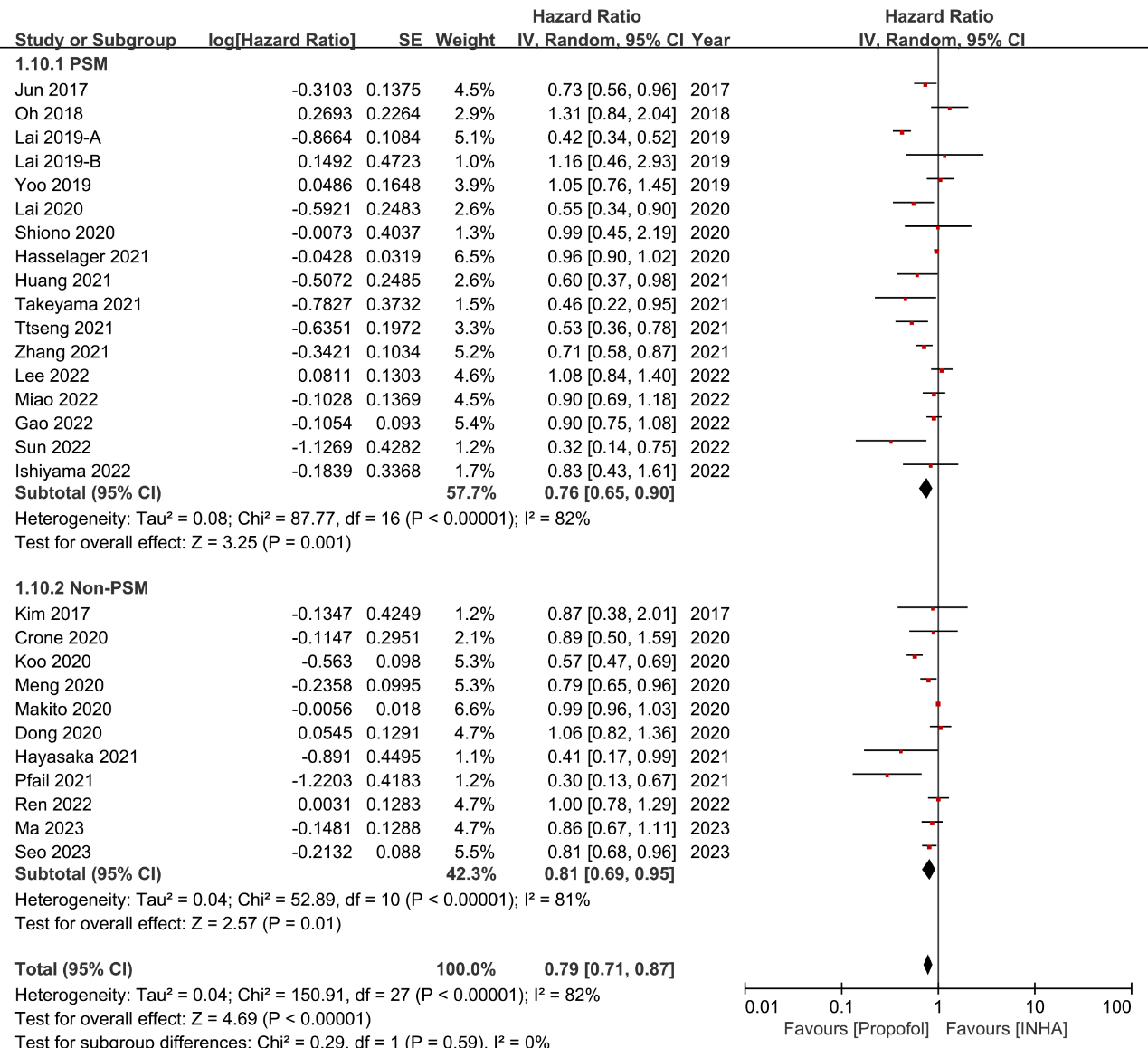


**Supplementary Table 1:** PRISMA 2020 checklist

| **Section and Topic** | **Item #** | **Checklist item** | **Location where item is reported** |
| --- | --- | --- | --- |
| **TITLE** | | |  |
| Title | 1 | Identify the report as a systematic review. | Title  Page 1 |
| **ABSTRACT** | | |  |
| Abstract | 2 | See the PRISMA 2020 for Abstracts checklist. | Abstract/ Paragraphs 1-4  Page 2-3 |
| **INTRODUCTION** | | |  |
| Rationale | 3 | Describe the rationale for the review in the context of existing knowledge. | Introduction/ Paragraphs 1-2  Page 4-5 |
| Objectives | 4 | Provide an explicit statement of the objective(s) or question(s) the review addresses. | Introduction/ Paragraph 2  Page 5 |
| **METHODS** | | |  |
| Eligibility criteria | 5 | Specify the inclusion and exclusion criteria for the review and how studies were grouped for the syntheses. | Methods/ Paragraph 3  Page 5-6 |
| Information sources | 6 | Specify all databases, registers, websites, organisations, reference lists and other sources searched or consulted to identify studies. Specify the date when each source was last searched or consulted. | Methods/ Paragraph 2  Page 5 |
| Search strategy | 7 | Present the full search strategies for all databases, registers and websites, including any filters and limits used. | Methods/ Paragraph 2  Supplementary Table S1  Page 5 |
| Selection process | 8 | Specify the methods used to decide whether a study met the inclusion criteria of the review, including how many reviewers screened each record and each report retrieved, whether they worked independently, and if applicable, details of automation tools used in the process. | Methods/ Paragraph 4  Page 6 |
| Data collection process | 9 | Specify the methods used to collect data from reports, including how many reviewers collected data from each report, whether they worked independently, any processes for obtaining or confirming data from study investigators, and if applicable, details of automation tools used in the process. | Methods/ Paragraph 5  Page 6 |
| Data items | 10a | List and define all outcomes for which data were sought. Specify whether all results that were compatible with each outcome domain in each study were sought (e.g. for all measures, time points, analyses), and if not, the methods used to decide which results to collect. | Methods/ Paragraph 5  Page 6-7 |
|  | 10b | List and define all other variables for which data were sought (e.g. participant and intervention characteristics, funding sources). Describe any assumptions made about any missing or unclear information. | Methods/ Paragraph 5  Page 6-7 |
| Study risk of bias assessment | 11 | Specify the methods used to assess risk of bias in the included studies, including details of the tool(s) used, how many reviewers assessed each study and whether they worked independently, and if applicable, details of automation tools used in the process. | Methods/ Paragraph 6  Page 7 |
| Effect measures | 12 | Specify for each outcome the effect measure(s) (e.g. risk ratio, mean difference) used in the synthesis or presentation of results. | Methods/ Paragraph 7  Page 7-8 |
| Synthesis methods | 13a | Describe the processes used to decide which studies were eligible for each synthesis (e.g. tabulating the study intervention characteristics and comparing against the planned groups for each synthesis (item #5)). | Methods/ Paragraph 3  Page 5-6 |
|  | 13b | Describe any methods required to prepare the data for presentation or synthesis, such as handling of missing summary statistics, or data conversions. | Methods/ Paragraph 7  Page 8 |
|  | 13c | Describe any methods used to tabulate or visually display results of individual studies and syntheses. | Methods/ Paragraph 7  Page 7-8 |
|  | 13d | Describe any methods used to synthesize results and provide a rationale for the choice(s). If meta-analysis was performed, describe the model(s), method(s) to identify the presence and extent of statistical heterogeneity, and software package(s) used. | Methods/ Paragraph 7  Page 8 |
|  | 13e | Describe any methods used to explore possible causes of heterogeneity among study results (e.g. subgroup analysis, meta-regression). | Methods/ Paragraph 7  Page 8 |
|  | 13f | Describe any sensitivity analyses conducted to assess robustness of the synthesized results. | None |
| Reporting bias assessment | 14 | Describe any methods used to assess risk of bias due to missing results in a synthesis (arising from reporting biases). | Methods/ Paragraph 7  Page 8 |
| Certainty assessment | 15 | Describe any methods used to assess certainty (or confidence) in the body of evidence for an outcome. | None |
| **RESULTS** | | |  |
| Study selection | 16a | Describe the results of the search and selection process, from the number of records identified in the search to the number of studies included in the review, ideally using a flow diagram. | Results/ Paragraph 1  (Figure 1) Page 8 |
|  | 16b | Cite studies that might appear to meet the inclusion criteria, but which were excluded, and explain why they were excluded. | Results/ Paragraph 1  (Figure 1) Page 8 |
| Study characteristics | 17 | Cite each included study and present its characteristics. | Results/ Paragraph 2  (Table 1) Page 8-9 |
| Risk of bias in studies | 18 | Present assessments of risk of bias for each included study. | Results/ Paragraph 3  (Table 2) Page 10 |
| Results of individual studies | 19 | For all outcomes, present, for each study: (a) summary statistics for each group (where appropriate) and (b) an effect estimate and its precision (e.g. confidence/credible interval), ideally using structured tables or plots. | Figure 3-4 |
| Results of syntheses | 20a | For each synthesis, briefly summarise the characteristics and risk of bias among contributing studies. | Results/ Paragraphs 5-14  Page 10-14 |
|  | 20b | Present results of all statistical syntheses conducted. If meta-analysis was done, present for each the summary estimate and its precision (e.g. confidence/credible interval) and measures of statistical heterogeneity. If comparing groups, describe the direction of the effect. | Results/ Paragraphs 5-14  Page 10-14 |
|  | 20c | Present results of all investigations of possible causes of heterogeneity among study results. | Results/ Paragraphs 5-14  Page 10-14 |
|  | 20d | Present results of all sensitivity analyses conducted to assess the robustness of the synthesized results. | None |
| Reporting biases | 21 | Present assessments of risk of bias due to missing results (arising from reporting biases) for each synthesis assessed. | Results/ Paragraph 4  Page 10 |
| Certainty of evidence | 22 | Present assessments of certainty (or confidence) in the body of evidence for each outcome assessed. | None |
| **DISCUSSION** | | |  |
| Discussion | 23a | Provide a general interpretation of the results in the context of other evidence. | Discussion/ Paragraph 1  Page 14 |
|  | 23b | Discuss any limitations of the evidence included in the review. | Discussion/ Paragraph 4  Page 17 |
|  | 23c | Discuss any limitations of the review processes used. | Discussion/ Paragraph 4  Page 17 |
|  | 23d | Discuss implications of the results for practice, policy, and future research. | Discussion/ Paragraph 2  Page 15-16 |
| **OTHER INFORMATION** | | |  |
| Registration and protocol | 24a | Provide registration information for the review, including register name and registration number, or state that the review was not registered. | Methods/ Paragraph 1  Page 5 |
|  | 24b | Indicate where the review protocol can be accessed, or state that a protocol was not prepared. | Methods/ Paragraph 1  Page 5 |
|  | 24c | Describe and explain any amendments to information provided at registration or in the protocol. | None |
| Support | 25 | Describe sources of financial or non-financial support for the review, and the role of the funders or sponsors in the review. | Funding  Page 18 |
| Competing interests | 26 | Declare any competing interests of review authors. | Conflict of Interest Statement  Page 18 |
| Availability of data, code and other materials | 27 | Report which of the following are publicly available and where they can be found: template data collection forms; data extracted from included studies; data used for all analyses; analytic code; any other materials used in the review. | Data availability statement  Page 18 |

*From:*  Page MJ, McKenzie JE, Bossuyt PM, Boutron I, Hoffmann TC, Mulrow CD, et al. The PRISMA 2020 statement: an updated guideline for reporting systematic reviews. BMJ 2021;372:n71. doi: 10.1136/bmj.n71

For more information, visit: <http://www.prisma-statement.org/>

**Supplementary Table 2:** Detailed search strategy

| **Database** | **Query** |
| --- | --- |
| **Pubmed** | #1 propofol [TIAB] OR total intravenous anesthesia [TIAB] OR propofol-based anesthesia [TIAB] OR TIVA [TIAB]  #2 inhalational anesthesia [TIAB] OR inhalation anesthesia [TIAB] OR volatile anesthesia [TIAB] OR sevoflurane [TIAB] OR desflurane [TIAB] OR isoflurane [TIAB] OR enflurane [TIAB] OR halothane [TIAB] OR INHA [TIAB]  #3 cancer [TIAB] OR tumor [TIAB] OR neoplasm [TIAB] OR carcinoma [TIAB] OR malignancy [TIAB]  #4 recurrence [TIAB] OR metastasis [TIAB] OR mortality [TIAB] OR survival [TIAB] OR long-term outcome [TIAB] OR long-term prognosis [TIAB] OR long-term consequence [TIAB]  #5 randomized controlled trial [all fields] OR observational study [all fields] OR retrospective study [all fields] OR prospective study [all fields]  #6 #1 AND #2 AND #3 AND #4 AND #5 |
| **Medline** | #1 propofol [TS] OR total intravenous anesthesia [TS] OR propofol-based anesthesia [TS] OR TIVA [TS]  #2 inhalational anesthesia [TS] OR inhalation anesthesia [TS] OR volatile anesthesia [TS] OR sevoflurane [TS] OR desflurane [TS] OR isoflurane [TS] OR enflurane [TS] OR halothane [TS] OR INHA [TS]  #3 cancer [TS] OR tumor [TS] OR neoplasm [TS] OR carcinoma [TS] OR malignancy [TS]  #4 recurrence [TS] OR metastasis [TS] OR mortality [TS] OR survival [TS] OR long-term outcome [TS] OR long-term prognosis [TS] OR long-term consequence [TS]  #5 randomized controlled trial [all fields] OR observational study [all fields] OR retrospective study [all fields] OR prospective study [all fields]  #6 #1 AND #2 AND #3 AND #4 AND #5 |
| **Embase and Cochrane library** | #1 propofol [ti,ab,kw] OR total intravenous anesthesia [ti,ab,kw] OR propofol-based anesthesia [ti,ab,kw] OR TIVA [ti,ab,kw]  #2 inhalational anesthesia [ti,ab,kw] OR inhalation anesthesia [ti,ab,kw] OR volatile anesthesia [ti,ab,kw] OR sevoflurane [ti,ab,kw] OR desflurane [ti,ab,kw] OR isoflurane [ti,ab,kw] OR enflurane [ti,ab,kw] OR halothane [ti,ab,kw] OR INHA [ti,ab,kw]  #3 cancer [ti,ab,kw] OR tumor [ti,ab,kw] OR neoplasm [ti,ab,kw] OR carcinoma [ti,ab,kw] OR malignancy [ti,ab,kw]  #4 recurrence [ti,ab,kw] OR metastasis [ti,ab,kw] OR mortality [ti,ab,kw] OR survival [ti,ab,kw] OR long-term outcome [ti,ab,kw] OR long-term prognosis [ti,ab,kw] OR long-term consequence [ti,ab,kw]  #5 randomized controlled trial [all fields] OR observational study [all fields] OR retrospective study [all fields] OR prospective study [all fields]  #6 #1 AND #2 AND #3 AND #4 AND #5 |

**Supplementary Table 3:** Characteristics of ongoing trails

| **Trail characteristics** | **Information** |
| --- | --- |
| **NCT01975064**  Design  Sponsor  Participants  Acutal enrollment  Interventions  Primary outcome measures  Secondary outcome measures  Start date  Trial status  Notes | **Cancer and Anesthesia: Survival After Radical Surgery - a Comparison Between Propofol or Sevoflurane Anesthesia (CAN)**  Phase4, Open-label, Multicenter, Randomized, Controlled Trial  Uppsala university  Patients older than 18 years who are scheduled for elective radical breast- or colorectal cancer surgery in general anesthesia. Radical surgery means that adjuvant treatment such as chemotherapy and/or radiation therapy seen as part of the curative treatment).  5574 participants  Sevoflurane/propofol  Overall survival [ Time Frame: Five years ]  Overall survival [ Time Frame: One year ]  November 2013  Completed  Information last updated on December 14, 2022; Actual completion date in August 31, 2022 |
| **NCT03034096** | **General Anesthetics in Cancer Resection Surgery (GA-CARES) Trial (GA-CARES)** |
| Design  Sponsor  Participants  Actual enrollment  Interventions  Primary outcome measures  Secondary outcome measures  Start date  Trail status  Notes | Phase 4, multicenter, randomized, double blinded study  Stony Brook University  Patients older than 18 years with known or suspected cancer and scheduled to undergo oncological surgical procedures.  1804 participants  sevoflurane, desflurane, or isoflurane/ propofol  All-cause mortality [Time Frame: 2 year minimum]  Recurrence-free survival [Time Frame: Minimum 2 years]  January 2017  Active, not recruiting  Information last updated in October 17, 2022; Estimated completion date on December 2024 |
| **NCT02756312**  Design  Sponsor  Participants  Estimated enrollment  Interventions  Primary outcome measures  Start date  Trail status  Notes | **The Impact of Anesthesia on High-Grade Glioma Patients**  Single-center, randomized, parallel group controlled clinical trial  Beijing Tiantan Hospital  Adult patients (18-80 years) with supratentorial high-grade glioma undergoing tumor resection  196 participants  Inhalation anesthesia/ intravenous anesthesia  overall survival in18 months [ Time Frame: Postoperative 18 months ]  October 1, 2020  Not yet recruiting  Information last updated on September 9, 2020; Estimated completion date on December 31, 2022 |
| **NCT04962672**  Design  Sponsor  Participants  Estimated enrollment  Interventions  Primary outcome measures  Secondary outcome measures  Start date  Trail status  Notes | **Anesthesia Induced Brain Cancer Survival (ABC Survival): A Feasibility Study**  Single-center, randomized, parallel assignment clinical trial  University Health Network, Toronto  Adult patients undergoing primary craniotomy for suspected high-grade glioma  40 participants  Sevoflurane/propofol  Rate of recruitment, retention rate, rate of protocol adherence [ Time Frame: 6 months ]  Overall survival and progression free survival [ Time Frame: 6 months ]  July 15, 2021  Recruiting  Information last updated on August 22, 2023; Estimated completion date on December 30, 2024 |
| **NCT05141877**  Design  Sponsor  Participants  Estimated enrollment  Interventions  Primary outcome measures  Secondary outcome measures  Start date  Trail status  Notes | **Influences of Propofol and Sevoflurane Anesthesia in Brain Tumor (anesthetics)**  Phase 4, Single-center, randomized, parallel assignment clinical trial  Kaohsiung Medical University Chung-Ho Memorial Hospital  Adult patients (20-80 years) undergoing elective craniotomy for primary brain tumors  706 participants  Sevoflurane/propofol  6-month, 1-year, and 3-year overall survival  The presence of disease progression [ Time Frame: up to 36 months]  December 2, 2021  Recruiting  Information last updated in October 31, 2023; Estimated completion date in November 30, 2025 |
| **NCT05606692**  Design  Sponsor  Participants  Estimated enrollment  Interventions  Primary outcome measures  Secondary outcome measures  Start date  Trail status  Notes | **Influences of Propofol and Sevoflurane Anesthesia in Ovarian Cancer**  Phase 4, Single-center, randomized, parallel assignment clinical trial  Kaohsiung Medical University Chung-Ho Memorial Hospital  Adult patients (20-80 years) undergoing elective craniotomy for primary ovarian tumors  416 participants  Sevoflurane/ propofol  progression-free survival [ Time Frame: 5 years ]  6-month, 1-year, 3-year, and 5-year overall survival   November 7, 2022  Recruiting  Information last updated in May 30, 2023; Estimated completion date in September 30, 2027 |
| **NCT03193710**  Design  Sponsor  Participants  Estimated enrollment  Interventions  Primary outcome measures  Secondary outcome measures  Start date  Trail status  Notes | **The Effects of General Anesthetics on Lymphocytes in Patients Undergoing Colorectal Cancer Resection and Mechanism Involved**  Single-center, randomized, prospective observational study  West China Hospital, Sichuan University  Adult patients (18-65 years) with colonal or rectal cancer undergoing cancer resection  260 participants  Sevoflurane/ propofol  Change from baseline lymphocytes within postoperative 5 years [Time Frame: up to 5 years]; cancer-free survival [Time Frame: 5 years or as available]  Cancer recurrence rate, cancer metastasis rate, concentration of cytokines up to 5 years, etc.  September 1, 2017  Recruiting  Information last updated in November 6, 2017; Estimated completion date in October 2023 |
| **NCT04513808**  Design  Sponsor  Participants  Estimated enrollment  Interventions  Primary outcome measures  Secondary outcome measures  Start date  Trail status  Notes | **Total Intravenous Anesthesia and Recurrence-free Survival After Esophageal Cancer Surgery**  Phase 3, Single-center, randomized, parallel assignment clinical trial  The Cleveland Clinic  Adult patients (19-90 years) undergoing Primary esophageal cancer surgery  950 participants  Sevoflurane/propofol  Recurrence-free survival [ Time Frame: 4 years ]  The treatment effect of propofol-based anesthesia versus volatile anesthesia. [ Time Frame: up to 2 day ]  August 14, 2020  Recruiting  Information last updated in November 15, 2023; Estimated completion date in December 2027 |
| **NCT****05343260**  Design  Sponsor  Participants  Actual enrollment  Interventions  Primary outcome measures  Secondary outcome measures  Start date  Trail status  Notes | **Impact of Anesthesia Maintenance Methods on 5-year Survival After Surgery**  Multicenter, randomized, controlled Trial  Peking University First Hospital  Elderly patients (65-90 years) who are scheduled to undergo surgery for the treatment of primary malignant tumor without receiving radiation therapy or chemotherapy preoperatively.  1228 participants  Sevoflurane/propofol  Over survival after surgery [ Time Frame: Five years ]  Recurrence-free survival and event-free survival after surgery [ Time Frame: Five years ]  April 1, 2015  Active, not recruiting  Information last updated in May 2, 2022; Estimated completion date in September 30, 2022 |
| **NCT05663242**  Design  Sponsor  Participants  Estimated enrollment  Interventions  Primary outcome measures  Secondary outcome measures  Start date  Trail status  Notes | **The Effects of Using Different Anesthetics on the Prognosis of Primary Lung Tumors and Its Mechanism of Action**  Single-center, randomized, parallel assignment clinical trial  Kaohsiung Medical University Chung-Ho Memorial Hospital  Adult patients (18-80 years) undergoing elective thoracic surgery for primary lung tumors  300 participants  Sevoflurane/propofol  6-month overall survival, 1-year overall survival, and 3-year overall survival; the presence of disease progression [ Time Frame: up to 36 months ]  Postoperative complications [ Time Frame: postoperative 30 days ]  December 27, 2022  Recruiting  Information last updated in May 30, 2023; Estimated completion date in November 30, 2026 |
| **NCT04259398**  Design  Sponsor  Participants  Actual enrollment  Interventions  Primary outcome measures  Secondary outcome measures  Start date  Trail status  Notes | **Anesthesia and Cancer Study: Colon Cancer**  Multi-center, prospective randomized, parallel assignment study  Seoul National University Hospital  Adults (19-80 years) undergoing surgery to remove colon cancer  797 participants  Sevoflurane/propofol  five year survival [ Time Frame: five years ]  1-year, 3-year, and 5-year recurrence-free survival; 1-year, and 3-year overall survival  February 18, 2020  Active, not recruiting  Information last updated in November 22, 2023; Estimated completion date in May 3, 2028 |
| **NCT****05331911**  Design  Sponsor  Participants  Estimated enrollment  Interventions  Primary outcome measures  Secondary outcome measures  Start date  Trail status  Notes | **Impact of Propofol-Based Total Intravenous Anesthesia Versus Anesthesia With Sevoflurane on Long-term Outcomes With Patients Undergoing Elective Excision of Primary Liver Tumors**  Phase 4, single-center, randomized, parallel assignment clinical trial  Kaohsiung Medical University Chung-Ho Memorial Hospital  Adult (20-80 years) patients undergoing partial hepatectomy for hepatocellular carcinoma  500 participants  Sevoflurane/propofol  6-month overall survival, 1-year overall survival, and 3-year overall survival; the presence of disease progression [ Time Frame: up to 36 months ]  Postoperative complications [ Time Frame: postoperative 30 days ]  April 26, 2022  Recruiting  Information last updated in November 30, 2022; Estimated completion date in March 31, 2027 |

Exclusion criteria, collaborators, and specific interventions are not shown, please consult the bellowed website for detailed information.

clinicaltrials.gov/ct2/show/NCT01975064, clinicaltrials.gov/ct2/show/NCT03034096, clinicaltrials.gov/ct2/show/NCT02756312, clinicaltrials.gov/ct2/show/NCT04962672, clinicaltrials.gov/ct2/show/NCT05141877, clinicaltrials.gov/ct2/show/NCT05606692, clinicaltrials.gov/ct2/show/NCT03193710, clinicaltrials.gov/ct2/show/NCT04513808,

clinicaltrials.gov/ct2/show/NCT05343260, clinicaltrials.gov/ct2/show/NCT05663242,

clinicaltrials.gov/ct2/show/NCT04259398, clinicaltrials.gov/ct2/show/NCT05331911.

,
